# Effects of short-term grazing exclusion on plant phenology and reproductive succession in a Tibetan alpine meadow

**DOI:** 10.1038/srep27781

**Published:** 2016-06-15

**Authors:** Juntao Zhu, Yangjian Zhang, Yaojie Liu

**Affiliations:** 1 Lhasa Plateau Ecosystem Research Station, Key Laboratory of Ecosystem Network Observation and Modeling, Institute of Geographic Sciences and Natural Resources Research, Chinese Academy of Sciences, Beijing, 100101, China; 2 CAS Center for Excellence in Tibetan Plateau Earth Sciences, Beijing 100101, China

## Abstract

Grazing exclusion (GE) has been widely considered as an effective avenue for restoring degraded grasslands throughout the world. GE, via modifying abiotic and biotic environments, inevitably affects phenological development. A five-year manipulative experiment was conducted in a Tibetan alpine meadow to examine the effects of GE on phenological processes and reproductive success. The study indicated that GE strongly affected phenological development of alpine plant species. Specifically, the low-growing, shallow-rooted species (LSS), such as *Kobresia pygmaea*, are more sensitive to GE-caused changes on upper-soil moisture and light. GE advanced each phonological process of *K. pygmaea*, except in the case of the treatment of fencing for 5 years (F5), which postponed the reproductive stage and lowered the reproductive success of *K. pygmaea*. Increased soil moisture triggered by GE, especially in the upper soil, may stimulate growth of LSS. However, the thick litter layer under the F5 treatment can influence the photoperiod of LSS, resulting in suppression of its reproductive development. These findings indicate that plant traits associated with resource acquisition, such as rooting depth and plant height, mediate plant phenology and reproductive responses to grazing exclusion treatments.

Grasslands cover approximately 40% of the earth’s terrestrial surface. They play a significant role in regulating and feedback to climates^1^,^2^. Globally, grazing by livestock is one of the most common human disturbances to grasslands. Overgrazing can cause severe grassland degradation^3^ and reduce both productivity and resilience^4^,^5^. After extensive practices, grazing exclusion (GE) has gradually been recognized as a simple, effective method for restoring degraded grasslands throughout the world^6^.

Recent research has focused on the effects of GE on vegetation succession, plant diversity, community structure and productivity^6^,^7^, and soil biogeochemical processes^8^,^9^. Vegetation development processes and reproductive success rates, as important aspects of plant phenology, have profound implications for ecological functions^10^. For example, the unfolding and coloring of leaves are processes closely related to the length of the vegetation growing season (GSL)^11^, which can substantially affect ecosystem processes such as carbon and water cycling^12^,^13^. To date, few studies have examined the effects of GE on plant phenology.

Abiotic environmental factors, such as temperature^14^, water (precipitation and soil moisture)^15^, photoperiod^16^ and soil nutrients^17^, combined with the biotic factors, such as plant life history^15^, influence phenological processes and reproductive success^18^. GE may alter phenology indirectly by modifying microclimates, such as soil nutrients^19^, soil moisture^20^, soil temperature^21^, and photoperiod^22^. For example, by encouraging a thicker litter layer, GE would decrease water loss through soil evaporation^23^, and possibly advance vegetation green-up. Although changes in abiotic factors can lead to significant consequences for phenological development, there is still a severe shortage of information on responses of phenological processes and reproductive success to GE, especially for the alpine grassland ecosystem.

Grasslands cover approximately 40% of the land in China^24^. Over the past 20 years, the Chinese government has invested heavily in the restoration of degraded grasslands. i For example, the ‘Start-up Re-grass Program’ on the Tibetan Plateau^25^ has been in place since 2004 to protect grasslands from heavy grazing through GE. Previous research in this region has focused on the impacts of GE on vegetation restoration and soil properties^26^,^27^. To enhance our understanding of the efficiency of GE treatments, knowledge of its effects on plant phenology are necessary. In order to meet this need, a five-year manipulative experiment was conducted in a Tibetan alpine meadow to examine the effects of GE treatment on phonological processes and reproductive success. Three major questions were addressed: (i) How does GE influence phenological timing and duration? (ii) What impacts does GE exert on plant reproductive success? (iii) What are the physiological mechanisms underlying the above two processes?

## Materials and Methods

### Study area

This experiment was conducted in a typical alpine meadow grassland at Naqu, northern Tibet, China (31°38.513′ N, 92°0.921′ E), at an approximate elevation of 4600 m. The mean annual temperature is −1.2 °C. The mean annual precipitation is 430 mm, and occurs mainly during the summer season from June to September. Winter precipitation, which typically falls as snow, is low in this region^15^. The growing season normally starts in mid-May and lasts until mid-September. The vegetation is dominated by *Kobresia pygmaea*, accompanied by *Potentilla saundersiana, Potentilla cuneata, and Stipa purpurea*.

### Study design

The GE manipulation treatment was started in 2010 using a chain link fence in a flat area covering approximately 1 × 1 km^2^. The GE treatments were arranged using a randomized block design, each block covering an area of 60 × 60 m^2^. By 2015, there were a total of six blocks. The treatments considered in this study include grazing (G); fenced for 1 year (F1); fenced for 2 years (F2); fenced for 3 years (F3); fenced for 4 years (F4); and fenced for 5 years (F5). Within each block, six 2 × 2 m^2^ plots in a diagonal direction, while avoiding block edges, were randomly delineated. On an overall basis, this design included 36 plots under six treatment levels, and six replicates for each treatment. While the experiment was conducted over a five-year period, all measurements were taken in 2015.

### Data collection

In one of the reference blocks, soil temperature (°C) and volumetric water content (%) were continuously monitored at a 5 cm depth in 2015 using Decagon EC-TM sensors (Decagon Devices, Pullman, Washington, USA). Two soil sensors were installed for each treatment, and the average of the two readings was recorded. Litter from six 30 × 30 cm^2^ subplots within each block was collected twice, during the early and late growing seasons. Collected litter was oven-dried at 65 °C to a constant weight. Litter layer depth (cm) was measured using a ruler. Soil samples were collected with a soil auger at three depths (0–10, 10–20 and 20–30 cm). Soil sampling was repeated three times for each block. 54 soil samples were rinsed from roots under running water over a 2-mm screen and dried at 105 °C for 12 h; their C and N concentrations were then measured using an Elementar Vario EL C/Nanalyzer (Elementar, Hanau, Germany). The soil P content was determined using the H_2_SO_4_-HClO_4_ fusion method.

*K. pygmaea, P. saundersiana, P. cuneata, S. purpurea*, and *Festuca coelestis* were selected as the focal species, whose coverage and biomass account for more than 90% of the community. In May, 2015, ten individuals of *K. pygmaea* and five individuals of other species were selected from each plot and then marked using a color-coded tag. The phenological processes of each selected individual were scored every 3–5 days using a scoring method modified from Dunne *et al*. (2003) and Xia *et al*.^28^,^29^. For forbs, the following codes were recorded: sprout-out leaf: 0; unopened buds: 1; opened flowers: 2; old flowers: 3; initiated fruits: 4; enlarged fruits: 5; dehisced fruits: 6; withered plants: 7. The following codes were applied to grasses: sprout-out leaf: 0; plant booting stage: 0.5; presence of spikelets: 1; exerted anthers or styles: 2; past the presence of anther and styles: 4; disarticulated florets: 6; and withered plants: 7. On each census day, unweighted averages of phenological scores for each individual plant were calculated^30^. For example, a plant with one bud (‘1’), three old flowers (‘3’), and four expanding fruits (‘5’) received a phenological score of 3.0^31^.

Based on their morphological and life-history traits, *K. pygmaea* were classified as either low-growing (3.2 cm height), shallow-rooted (10 cm depth) or early-flowering species (DOY: 155 ± 0.8). In a similar vein, *P. saundersiana* and *P. cuneata* were classified as low-growing (4.0 cm height), shallow-rooted (10 cm depth) or mid-flowering species (DOY: 168 ± 0.4; DOY: 172 ± 1.1). Finally, *S. purpurea* and *F. coelestis* were classified as tall-growing (25 cm height), deep-rooted (30 cm depth) or late-flowering species (DOY: 193 ± 0.5; DOY: 197 ± 1.2; DOY: 208 ± 0.4).

### Data analysis

It is most difficult to directly obtain the exact timing of flowering and fruiting on the basis of observations taken at 3–5 day intervals^29^. Usually these data are extracted by fitting the observed data to statistical models, such as a linear regression model^30^, or the Richards growth equation. For example, the latter has been successfully applied to derive daily plant phenology in a tallgrass prairie in North America^32^ and a semi-arid temperate steppe in Inner Mongolia, China^29^. The relevant equation is:


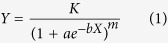


where *K* is the maximum growth; *a* is a parameter related to the first observation date; *b* is the growth rate over time *X* in days; and *m* is a parameter related to the curve shape^29^. The timing of each phenological event can be calculated from Equation 1 as:


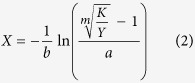


In this study, the Richards equation with the contraction–expansion algorithm was applied to fit phenological scores (*Y*) of each species against the day for each plot; this was accomplished utilizing Matlab (Mathworks, Natick, MA, USA). Best parameter estimates of *K, a, b* and *m* were obtained for each species. Equation (2) was applied to calculate the vegetative timing (i.e., green-up, ‘0’), reproductive timing (i.e., flowering and fruiting, ‘2’ and ‘3.5’), and the growing season length (GSL).

### Statistical analysis

A two-way analysis of variance (ANOVA) with Turkey’s test was applied to disentangle the effects of the plant species (*K. pygmaea, P. saundersiana, P. cuneata, S. purpurea*, and *Festuca coelestis*) and treatments for grazing (G) and grazing exclusion (F5, F4, F3, F2, F1) on green-up, flowering, timing of fruiting, and growing season length. A one-way ANOVA with Tukey’s HSD test were used to analyze the effects of grazing and grazing exclusion treatments on litter depth, litter weight, soil conditions, phenological stages and reproductive success. All comparisons were considered significantly different at *P* < 0.05. All statistical tests were run using SPSS (v. 19.0; Chicago, USA).

## Results

### Microclimate, litter layer, and soil nutrients

**Figure 1:**
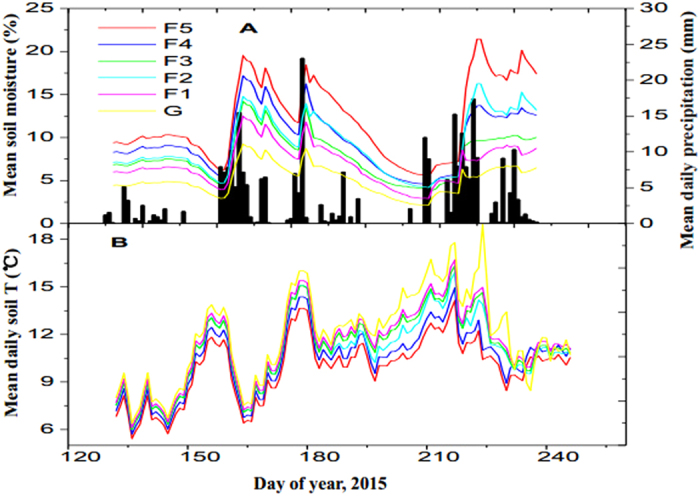
Mean soil moisture (%) at 5 cm depth [**A**], soil temperature at 5 cm depth (°C) [**B**] and daily precipitation (mm) [**A**] during the growing season under different fencing treatments in 2015. F5, F4, F3, F2, F1, and G represent plots with fencing for 5, 4, 3, 2, and 1 year (s) and grazing, respectively.

**Figure 2:**
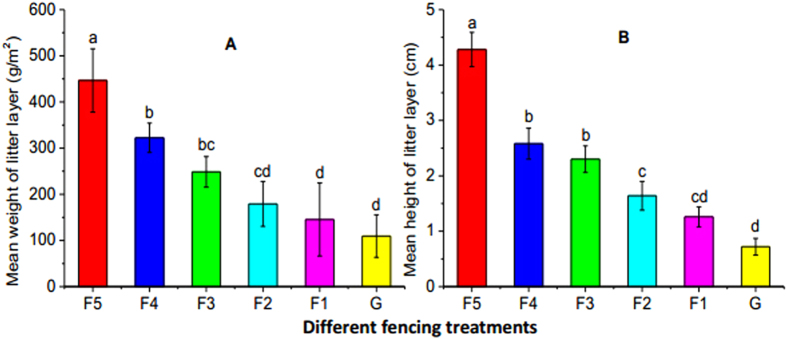
Mean weight (**A**) and depth (**B**) of the litter layer under different fencing treatments during the growing season of 2015. Different letters indicate significant differences at 5% level among treatments. F5, F4, F3, F2, F1, and G represent plots with fencing for 5, 4, 3, 2, and 1 year (s) and grazing, respectively.

The consequences of each fencing treatment were compared to the grazing control treatment. The F5, F4, F3, F2, and F1 treatments increased growing season mean soil moisture at 5 cm depth by 6.0, 4.9, 3.5, 2.2, 1.7%, respectively (Fig. 1A). The F5, F4, F3, F2, and F1 decreased mean soil temperature at 5 cm depth by 1.5, 1.1, 0.8, 0.6, and 0.5 °C, respectively (Fig. 1B). The F2 and F1 treatments did not significantly change the amount of litter (Fig. 2A, *P* > 0.05). The F5, F4, and F3 treatments significantly increased the litter layer weight by 308%, 195% and 127%, respectively (Fig. 2A, *P* < 0.05). Compared to the grazing treatment, grazing exclusion (GE) significantly increased litter layer depth (*P* < 0.05), except for F1 (Fig. 2B, *P* > 0.05).There were significant differences in litter layer weight and depth between treatments F5 and F4, F3 (Fig. 2, *P* < 0.05).The five fencing treatments caused no significant effects on total soil content of C, N, P or the C/N ratio (Table 1, *P* > 0.05).

### Phenological stages

**Figure 3:**
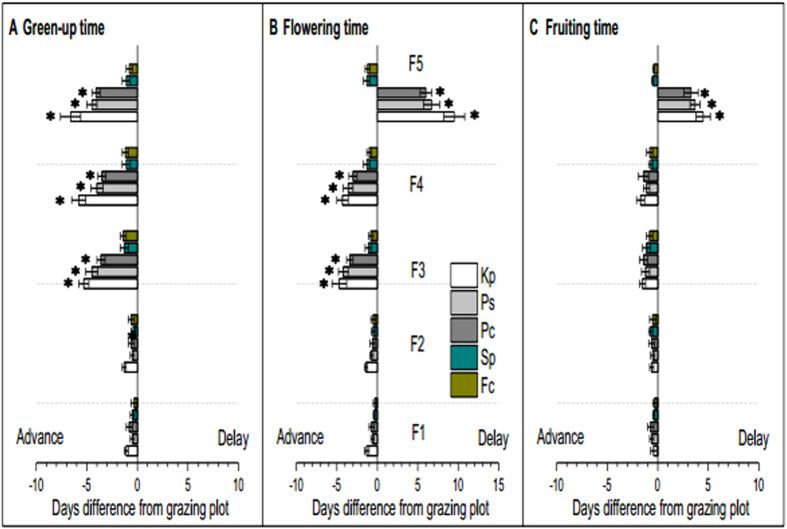
Changes (in days) in the green-up (**A**), flowering (**B**) and fruiting (**C**) times for five fencing treatments compared with the grazing plot in 2015. A positive value indicates later green-up, flowering or fruiting than that in the grazing plot; while a negative value indicates earlier green-up, flowering or fruiting than in the grazing plot. Data are mean ± SE for advanced or delayed phenology. ^*^ indicates significant differences at the 5% level between grazing exclusion and grazing treatments. F5, F4, F3, F2, F1, and G represent plots with fencing for 5, 4, 3, 2, 1 years and grazing, respectively. Kp, Ps, Pc, Sp, Fc represent *Kobresia pygmaea, Potentilla saundersiana, Potentilla cuneata, Stipa purpurea* and *Festuca coelestis*, respectively.

**Figure 4:**
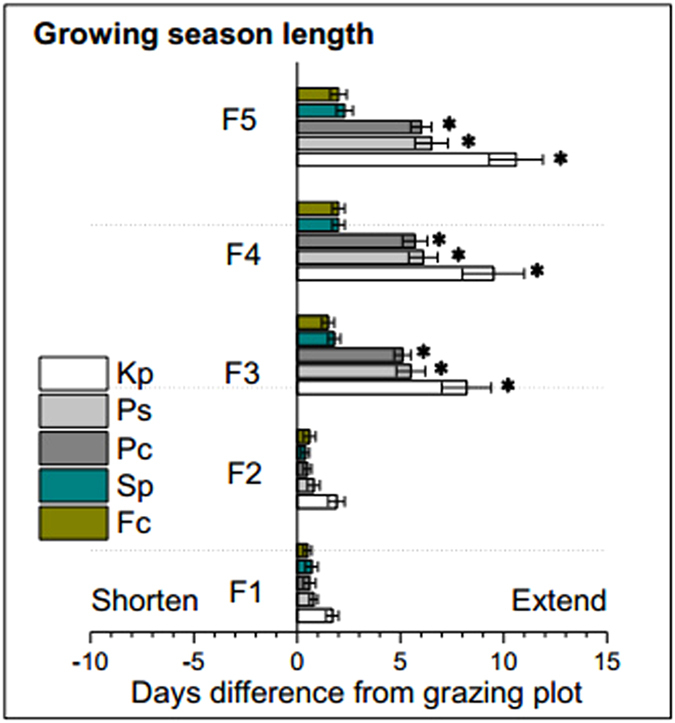
Changes in the growing season length (GSL) in five fencing treatments compared to grazing plot in 2015. Negative values (−) indicate shortened a GSL and positive values (+) indicate an extended GSL. Data are mean ± SE for shortened or extended days. ^*^ indicate significant differences at 5% level between grazing exclusion and grazing treatments. F5, F4, F3, F2, F1, and G represent plots with fencing for 5, 4, 3, 2, 1 years and grazing, respectively. Kp, Ps, Pc, Sp, Fc represent *Kobresia pygmaea, Potentilla saundersiana, Potentilla cuneata, Stipa purpurea* and *Festuca coelestis*, respectively.

The results of two-way ANOVAS showed that the grazing exclusion (GE) treatments and plant species both exerted significant effects on the green-up, flowering and fruiting time, as well as length of growing season length (Table 2, *P* < 0.01), also significant interactive effects on the phenological events (Table 2, *P* < 0.01). For the phonological events, consequences of each fencing treatment were compared only with the control grazing treatment. The GE did not significantly affect plant phenology of *Sp* and *Fc* (Fig. 3A, *P* > 0.05). The F1 and F2 treatments did not significantly change plant phenology of any of the five selected species (Fig. 3, *P* > 0.05). The F3, F4 and F5 treatments significantly advanced the green-up dates for *Kp, Ps*, and *Pc* (Fig. 3A, *P* < 0.05). The F3 and F4 treatments significantly advanced the flowering dates for *Kp, Ps*, and *Pc* (Fig. 3B, *P* < 0.05). In contrast, the F5 treatment significantly delayed the flowering dates for *Kp, Ps*, and *Pc* by 9.5 (±1.3 SE), 6.7 (±1.0) and 6.0 (±0.7) days, respectively (Fig. 3B, *P* < 0.05). The GE treatments did not significantly change the fruiting date for any of the five species (Fig. 3C, *P* > 0.05), with the notable exception of F5, which significantly delayed the fruiting date in *Kp, Ps*, and *Pc* by 4.5 (±0.7), 3.7 (±0.5) and 3.3 (±0.7) days, respectively (Fig. 3C, *P* < 0.05). With respect to growing season length (GSL), the F1 and F2 treatments did not significantly change the GSL for any of the five species (Fig. 4, *P* > 0.05). The F3, F4 and F5 significantly extended the GSL of *Kp, Ps*, and *Pc* (Fig. 3A, *P* < 0.05). For example, the GSL of *Kp* was extended by 8.2 (±1.2), 9.5 (±1.5) and 10.6 (±1.3) days under treatments F3, F4 and F5, respectively (Fig. 4, *P* < 0.05).

### Reproductive success

**Figure 5:**
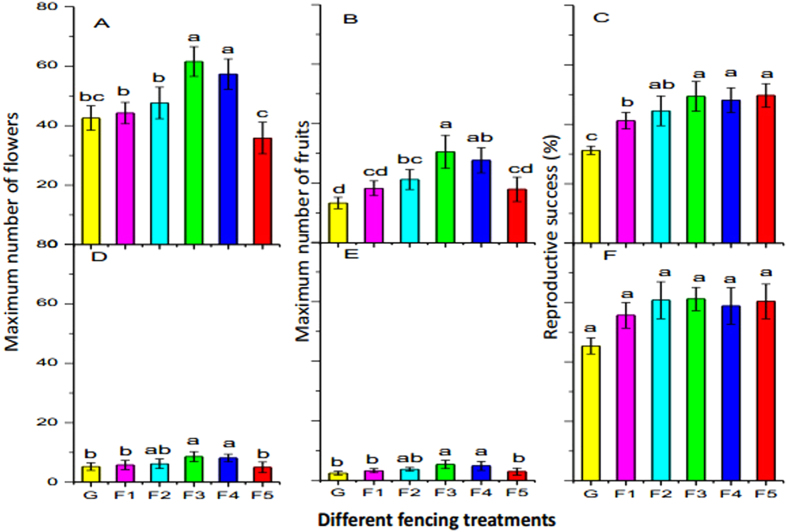
Effects of six experimental treatments on the maximum number of flowers, fruits and reproductive success of *Kobresia pygmaea* (**A**–**C**), *Potentilla saundersiana* (**D**–**F**) in 2015. Data are mean ± SE. Different letters indicate significant differences at the 5% level among treatments. F5, F4, F3, F2, F1, and G represent plots with fencing for 5, 4, 3, 2, 1 years and grazing, respectively.

Compared to the grazing treatment, the F1, F2 and F5 treatments did not significantly affect the maximum number of flowers in *K. pygmaea* and *P. saundersiana* (Fig. 5A,D, *P* > 0.05), while the F3 and F4 significantly increased the number of flowers of these two species (Fig. 5A,D, *P* < 0.05).The maximum number of fruits in *K. pygmaea* and *P. saundersiana* was higher under the F3 and F4 treatments than under the grazing treatment (Fig. 5B,E, *P* < 0.05). However, the F5 treatment significantly reduced the maximum number of fruits in *K. pygmaea* and *P. saundersiana* compared with treatments F3 and F4 (Fig. 5B,E, *P* < 0.05). Exclusion of grazing (GE) significantly increased reproductive success for *K. pygmaea* (Fig. 5C, *P* < 0.05). The reproductive success of *P. saundersiana* was not affected by any treatment when compared to the grazing treatment (Fig. 5F, *P* > 0.05).

## Discussion

### Microclimate, litter layer, and soil nutrients

Decomposition of plant litter is a key process in nutrient and carbon cycling for terrestrial ecosystems^33^. The litter decomposition in cold biomes, such as alpine meadows, is strongly limited by temperature^34^,^35^. As shown in the present study, the weight and depth of the litter layer increased with the length of the grazing exclusion period. Thus by thickening the litter layer, grazing exclusion lowered water loss from soil evaporation^23^, and ameliorated upper-soil moisture conditions. Soil temperature in the grazing exclusion plots was lower than that in the grazing plots, which is in line with previous studies on the Tibetan plateau^35^. Grazing exclusion has been considered to be an effective management practice capable of boosting soil C and N contents in rangelands^36^. This study found that grazing exclusion failed to alter total C, N, P content in the soil as well as the C/N ratio^8^, which might be a consequence of the short period of grazing exclusion treatment in this work. In the northern Tibetan grasslands, temperature is extremely low and litter decomposes slowly. As a result, a large proportion of the enriched litter in grazing-excluded sites have not as yet entered the soil after several years of grazing exclusion.

### The timing of green-up

The grazing exclusion treatments, such as F3, F4 and F5, advanced the green-up date for low-growing, shallow-rooted species. In the alpine meadows of Northern Tibet, these species normally turned green in late May, a dry and pre-monsoon period. The ameliorated soil moisture condition^23^, especially in the upper soil, under grazing exclusion treatment may stimulate growth of low-growing, shallow-rooted plants such as *Kp, Ps*, and *Pc*. This is in accord with the previous findings that the shallow-rooted plants in the northern Tibetan plateau are more sensitive to upper-soil moisture^15^,^37^. In addition, spring phenology responses to climate change appear not to be strongly constrained by photoperiod^38^. Grazing exclusion, via a thickened litter layer, may influence photoperiod^16^,^22^, but in this study it had no effect on the timing of green-up. Tall-growing, deep-rooted species, such as *Sp* and *Fc*, are able to utilize deep soil water. Their phenological development is less constrained by water availability compared to that of low-growing, shallow-rooted species^15^. The increased soil moisture under grazing exclusion may not reach the level to stimulate the phenological processes of the deep-rooted species.

### The timing of flower and fruit

Grazing exclusion treatments, such as F3 and F4, advanced the flowering date for the low-growing, shallow-rooted species. Through thickening of the litter layer, grazing exclusion increased soil moisture, resulting in accelerated plant growth, and consequently advancing the flowering date^39^. Soil temperature in the grazing exclusion plots was lower than that of the grazing plots during the growing season, yet phenology was still advanced under treatments F3 and F4. Thus soil moisture, rather than soil temperature, was likely the major phenological cue for the alpine grassland in Northern Tibet^15^,^40^. The process of producing fruit follows several other predecessor processes. Although the green-up and flowering times of low-growing, shallow-rooted species were advanced under the F3 and F4 treatments, plants may compensate for changes in individual phenological events. As a result, the fruit timing was unaltered^41^.

Plant phenology is affected by a variety of climatic factors, including humidity^15^ (precipitation and soil moisture), photoperiod^16^, temperature^14^ and winter chilling^42^. Interestingly, the flowering and fruiting responses of the low-growing, shallow-rooted species to the grazing exclusion period were reversed for the fifth year of grazing exclusion. Photoperiod responses to plant phenology are driven by the circadian clock^43^, and these responses may serve as a buffer to avoid, for example, the immediate response of phenology to temperature. This acclimation might be a potential reason for the reversed responses of plant phenology for the fifth year of grazing exclusion. Another reason why the pattern was reversed under the F5 treatment may be decreased radiation due to a thickened litter layer^44^. The low-growing, shallow-rooted species are buried under the litter layer, especially by the fifth year of grazing exclusion. The tall-growing, deep-rooted species can utilize their height advantage to spike through the litter layer. Thus their reproductive development is not constrained by photoperiod and radiation amount. The differentiated phenology responses to the grazing exclusion period gradient illustrated that the photoperiod and radiation level are the main factors regulating plant reproductive growth under grazing exclusion treatments.

### The growing season length and reproductive success

Climate warming has been reported to extend the length of the growing season^32^,^45^, principally through an earlier beginning or later termination thereof^46^. For the alpine meadow ecosystem in Northern Tibet, grazing exclusion extended growing season lengths (GSLs) of low-growing, shallow-rooted species by advancing their green-up timing, which is consistent with findings for Northern Tibet based on remote sensing^47^.

The grazing exclusion treatments, such as F3 and F4, increased the number of flowers and fruits in *K. pygmaea* and *P. saundersiana*. Dorji *et al*. (2013) have reported that warming, via decreasing soil moisture, can suppress the reproductive effort of *K. pygmaea*^15^. Conversely, grazing exclusion, via increased soil moisture, particularly in the upper soil, can stimulate the reproductive efforts of low-growing, shallow-rooted species in Northern Tibet. The F3 and F4 treatments also significantly increased reproductive success in *K. pygmaea*. Such improved success resulted mainly from the combined effects of changes in soil moisture and presence of grazing animals^31^. However, grazing exclusion failed to alter the reproductive success rate of *P. saundersiana*. Previous studies have reported that feeding selection by livestock can influence species composition and community structure^48^. In Northern Tibet, grazing livestock tend to feed on species of Cyperaceae (*K. pygmaea*) and Gramineae (*Stipa purpurea*). Grazing exclusion, through removal of livestock, would be more likely to increase reproductive success of *K. pygmaea*, while having less (or in the case of this study, no) effects on forbs such as *P. saundersiana*.

Despite increased soil moisture, the F5 treatment significantly reduced the maximum number of flowers, fruits and reproductive success for *K. pygmaea* and *P. saundersiana* compared to the F3 and F4 treatments. Photoperiodic constraints on plant phenology can also affect photosynthetic activity of plants^49^. In northern Tibet, the F5 treatment, via an increased litter layer, can influence the photoperiod^16^,^22^ and photosynthetic activity of the low-growing, shallow-rooted species, further suppressing their reproductive development and consequently reducing the number of flowers, fruits and reproductive success.

Grazing exclusion strongly affected alpine plant phenology and reproductive success in the focal alpine system of this study. Species traits, such as low- vs. tall growing and shallow vs. deep rooting depth, can mediate how alpine plant species respond to grazing exclusion. The low-growing, shallow-rooted species, such as *K. pygmaea*, are more sensitive to grazing exclusion due to changes in upper-soil moisture and light resources. Grazing exclusion generally advanced the phenology of *K. pygmaea*, yet exclusion for five years delayed the reproductive timing and success of *K. pygmaea* via thickening of the litter layer and effect on the photoperiod. The tall-growing, deep-rooted species can spike through the litter layer due to their height advantage. Thus their reproductive development is not constrained by light limitations.

Related studies have reported that grazing exclusion can decrease species diversity and soil organic C sequestration in our targeted alpine system^26^,^50^. This study provided evidence that three and four years of grazing exclusion can advanced the phenology of low-growing, shallow-rooted species, while exclusion of grazing for five years can postpone their reproductive timing. Thus, three to four years of grazing exclusion may be an efficient way to restore degraded grasslands in the Tibetan alpine meadow. As we did not directly investigate light quantity and photoperiod under GE treatments, results of this study were not able to distill out the effects of photoperiod on phenological development. Thus, future work is required to examine the sole effect of photoperiod on plant phenology under grazing exclusion measures.

## Additional Information

**How to cite this article**: Zhu, J. *et al*. Effects of short-term grazing exclusion on plant phenology and reproductive succession in a Tibetan alpine meadow. *Sci. Rep.* **6**, 27781; doi: 10.1038/srep27781 (2016).

## Figures and Tables

**Table 1:** Soil C, N, and P content and CN ratio for plots with fencing for 5, 4, 3, 2, and 1 year (s) and grazing plot. Data points show means ± SE.

Treatments	N (%)	C (%)	P (mg/g)	CN Ratio
F5	0.23 ± 0.07	2.44 ± 0.94	0.76 ± 0.04	10.16 ± 0.87
F4	0.24 ± 0.06	2.48 ± 0.69	0.76 ± 0.05	10.24 ± 0.35
F3	0.25 ± 0.06	2.37 ± 0.76	0.74 ± 0.05	9.42 ± 0.57
F2	0.22 ± 0.07	2.32 ± 0.93	0.72 ± 0.04	9.89 ± 0.93
F1	0.24 ± 0.07	2.43 ± 0.93	0.72 ± 0.05	9.82 ± 0.70
G	0.25 ± 0.07	2.59 ± 0.87	0.75 ± 0.04	10.21 ± 0.59

Different letters indicate significant differences at 5% level among treatments.

**Table 2:** Results (*F* Values) of two-way ANOVA on the effects of grazing exclusion treatments (GE), plant species and their interactions on the green-up, flowering and fruiting time and the growing season length of five selected grassland species.

Source	*DF*	Green-up time		Flowering time	
		***F***	***P***	***F***	***P***
GE	4	55.627	0.000	130.398	0.000
Species	5	1014.253	0.000	6981.986	0.000
GE × Species	20	5.378	0.000	25.569	0.000
		**Fruiting time**		**Growing season length**	
GE	4	24.559	0.000	78.803	0.000
Species	5	5819.444	0.000	363.652	0.000
GE × Species	20	3.551	0.000	6.684	0.000
